# The feasibility of a Two-incision video-assisted thoracoscopic lobectomy

**DOI:** 10.1186/1749-8090-8-88

**Published:** 2013-04-15

**Authors:** Hyun Koo Kim, Ho Kyung Sung, Hyun Joo Lee, Young Ho Choi

**Affiliations:** 1 Departments of Thoracic and Cardiovascular Surgery, Korea University Guro Hospital, Korea University College of Medicine, 97 Guro-donggil, Seoul, Guro-gu 152-703, Korea

**Keywords:** Thoracosocopy/VATS, Lobectomy

## Abstract

**Background:**

This study is to evaluate the feasibility and safety of video-assisted thoracoscopic (VATS) lobectomy with two incisions.

**Methods:**

A total of 73 patients (male 47, female 26; mean age 61.2 ± 12.00 years old) who underwent major pulmonary resection, through VATS, using two incisions were included in this study. The thoracoscopy port was placed at the 7th or the 8th intercostal space in the mid-axillary line, and the working port, 3~5 cm long, at the 5th intercostal space, on the operator’s side.

**Results:**

The preoperative diagnosis was benign lung disease in 8 patients (11.0%) and malignant lung disease in 65 (89.0%). Two patients (3.1%) needed a third port during surgery due to severe pleural adhesion, and conversion to thoracotomy was needed in 5 (6.8%), due to bleeding at pulmonary arterial branch (n = 3), anthracofibrotic lymph nodes around pulmonary artery (n = 1), and severe pleural adhesion (n = 1). The mean duration of the operation in the 66 patients, completed by a two-incision VATS lobectomy, was 163.4 ± 30.40 minutes. In 56 cases, which were completed by a two-incision VATS lobectomy for primary lung cancer, a total number of dissected lymph nodes per patient were 20.2 ± 11.2. The chest tube was removed on postoperative day 5.4 ± 2.8, and there was no occurrence of major perioperative morbidity and mortality.

**Conclusions:**

Two-incision VATS lobectomy is applicable in the selected cases, and may obtain similar results with the conventional VATS lobectomy, through a certain period of learning curve.

## Background

Since the first video-assisted thoracic surgery (VATS) lobectomy, with anatomic hilar dissection performed in 1992, the frequency of this procedure has increased due to its attractiveness as a minimally invasive modality, which results in fewer postoperative complications, and reduces the duration of pleural drainage, as well as a reduction in the length of the hospital stay [1-5]. However, the use of VATS lobectomy to perform anatomic lung resection for lung cancer remains controversial because there have been questions whether it reduces local disease recurrence and its positive effects of long-term survival [6,7]. Although a recent meta-analysis of randomized and nonrandomized trials demonstrated that VATS lobectomy is an appropriate procedure for selected patients with early-stage non-small cell lung cancer (NSCLC), when compared with open surgery [8,9], more sufficient information regarding the oncologic efficacy of this procedure needs to accumulate to gain wide spread acceptance of this procedure [5,10].

Nevertheless, the literature shows that in the hands of experienced VATS surgeons, a lobectomy is a safe operation that offers patients comparable or better complication rates, compared to that of the conventional lobectomy by thoracotomy [11]. Moreover, as VATS lobectomy techniques continue to improve by some pioneers in the field of VATS lobectomy, attempts to decrease the size of the working port, the diameter of thoracoscope and instruments, as well as the number of incision has gradually been made [12-15]. Recently, Borro and colleagues [15] reported the feasibility of VATS lobectomy with two ports and said that the 3rd port is not necessary for the majority of cases, despite the use of three ports by most of the surgeons.

Although the third port was already made at the beginning of the operation, we occasionally did not use this port during a VATS lobectomy procedure. This is because, on a usual case, the operator only used the working port, which was made at the operator’s side, and the assistant therefore, used the thoracoscopic port. The 3rd port, which was made at the assistant side, was usually unnecessary during a VATS lobectomy with the exception of some unexpected situations. Therefore, the author (K.H.K) started to perform VATS lobectomy with only two working ports, without the 3rd port, since July 2010. In this study we evaluated the safety and feasibility of VATS lobectomy with two incisions.

## Methods

Consecutive patients, who underwent major pulmonary resection (segmentectomy, lobectomy, bilobectomy, and pneumonectomy) through VATS using two incisions, from July 2010 to December 2011, in Korea Unversity Guro Hospital, were included in this study. This study was performed through a retrospective chart review. It was approved by the Korea University Guro Hospital Ethics Committee (KUGH12076) and the informed consent was waived.

As long as lesions were amenable to anatomic resection and patients were expected to be able to tolerate single-lung ventilation, as determined by preoperative pulmonary function tests, we performed a two-incision VATS lobectomy to various benign or malignant pulmonary diseases [10]. For benign lung diseases, VATS lobectomy was not attempted when preoperative computed tomography (CT) findings revealed the following: a definite pleural calcification, tight calcification stuck to the pulmonary vessel, thoracic cage deformity, or decreased volume of ipsilateral lung, which all implied severely dense adhesion [10]. For malignant lung disease, absolute contraindications include a tumor of 6 cm or larger, invasive mediastinal tumor, and the inability to perform a complete resection with lobectomy, bilobectomy, or pneumonectomy. Relative contraindications include advanced disease or neoadjuvant chemo- or radiotherapy, endobronchial tumor requiring bronchoplasty, or lymph node affectation, which seriously hinders hilar dissection.

**Figure 1:**
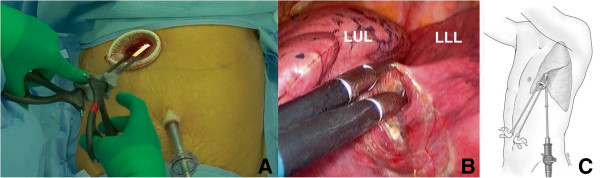
**Two-incision video-assisted thoracoscopic (VATS) left lower lobectomy. **(**A**) The operator always took place at the right side of the patient, a thoracoscopy port is located at the 7th intercostal space in the mid-axillary line, and a utility incision, 4 cm long, at the 5th intercostal space, in the operator side. (**B**) Dissection of major pulmonary fissure using 5 mm sized instruments through a utility incision. (**C**) Schematic illustration of a two-incision VATS left lower lobectomy.

**Figure 2:**
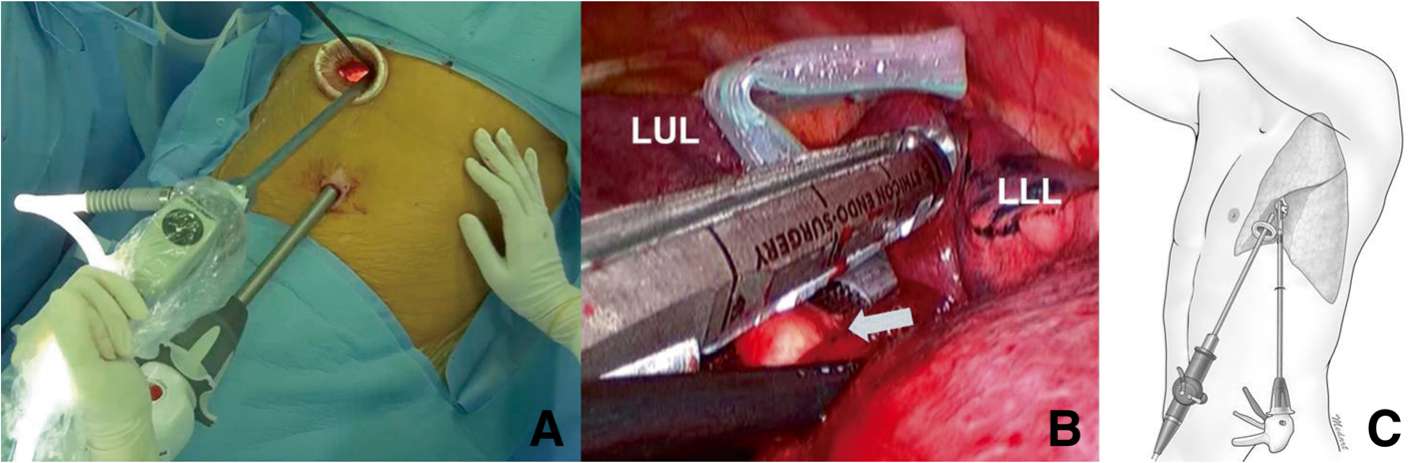
**Division pulmonary arterial branches to left lower lobe (A), (B), (C). **Exchanging the thoracoscope and the endostapler from one incision to the other during the two-incision video-assisted thoracoscopic left lower lobectomy. An arrow indicates pulmonary arterial branches to left lower lobe.

In our hospital, since VATS lobectomy launched in March 2006, two ports and a utility incision had been used without rib spreading. A 12 mm sized trocar, for the thoracoscope with 10 mm in size and 30 degree, was placed at the 7th or the 8th intercostal space in the mid-axillary line, and a 3 to 5 cm sized utility incision was made at the 5th intercostal space in the anterior or the posterior axillary line. An additional 5 mm sized trocar was placed at the 5th or the 6th intercostal space in the anterior or the posterior axillary line. In this study, we apply a two-incision VATS lobectomy without this 3rd port at any lobe from the start (Figure 1). The vessels and bronchi of the target lobe were individually dissected. For patients with benign disease, we prefer to staple the pulmonary artery first, and then the vein, whenever possible, to prevent vein congestion. For patients with malignant disease, we prefer to staple the pulmonary vein first and then the artery, whenever possible, to prevent possible tumor seeding through the pulmonary vein, and complete mediastinal lymph node dissection was mandatory. During this procedure, in case of needing assistant’s retraction of tissue, two instruments from the operator and one instrument from the assistant are usually introduced all together, through the working port. Further, we free to exchange the thoracoscope and the endostaplers from one incision to the other for the resection of hilar structures (Figure 2). All specimens were placed into an impermeable bag and removed through the utility incision, and a single chest tube is placed at the end of the surgery.

Variables, such as patient’s age and sex, histology, type and duration of surgery, duration of chest tube, length of hospital stay, and postoperative complications and mortality were analyzed. In addition, for patients with cancer, clinical and pathologic TNM stage of cancer, and the number of dissected lymph node were studied, additionally.

Descriptive statistics were used to describe the patient’s demographic characteristics and outcomes. Normally distributed continuous data were expressed as the mean ± standard deviation. Categorical data were expressed as count and proportion. Student’s t-test and the chi-square test or Fisher’s exact test were used to compare continuous and categorical variables, respectively. SPSS (ver. 12; SPSS Inc., Chicago, IL, USA) for windows was used for statistical analysis.

## Results

A total of 73, out of 108 (68%) (male 47, female 26; mean age 61.2 ± 12.00 years old, range 19~85), who underwent major pulmonary resection (segmentectomy, lobectomy, bilobectomy, or pnemonectomy) through a consecutive VATS using two incisions from July 2010 to December 2011, were included in this study. Among the remaining 35 patients, five patients, who had undergone neoadjuvant chemotherapy (n = 4) or concurrent chemoradiotherapy (n = 1), underwent a standard VATS lobectomy from the start, and they were excluded in this study. Therefore, 78 patients, out of 108 (72%), who underwent major pulmonary resection have undergone a VATS lobectomy.

The preoperative diagnosis was benign lung disease in 8 patients (11%) (3 pulmonary sequestration, 3 pulmonary tuberculosis, 1 emphysema, and 1 bronchogenic cyst) and malignant lung disease in 65 patients (89%) (61 primary lung cancer, 4 metastatic lung cancer) (Table 1). Clinical stage of primary lung cancer (n = 61) was T1 or T2N0M0 in 47 patients (77%), T3N0M0 in 3 (5%), and N1 (n = 9) or N2 (n = 2) in 11 (18%). Two patients with N2 disease, one with N1, and one with T3 underwent preoperative adjuvant chemotherapy.

**Table 1:** Patients’ characteristics

**Characteristics**				**No. (%)**
Histology (n = 73)	Malignancy			65 (89)
		Primary		61 (84)
			Adenocarcinoma	35 (48)
			Squamous cell carcinoma	18 (25)
			Others	8 (11)
		Metastasis		4 (6)
			Recurrent lung cancer (adenocarcinoma)	2 (3)
			Rectal cancer (Adenocarcinoma)	2 (3)
	Benign			8 (11)
		Tuberculosis		3 (4)
		Bronchiectasis		3 (4)
		Pulmonary sequestration		1 (1)
		Bronchogenic cyst		1 (1)
Type of surgery (n = 74)	Segmentectomy			2 (3)
	Right upper lobectomy			20 (27)
	Right middle lobectomy			5 (7)
	Right lower lobectomy			18 (24)
	Left upper lobectomy			11 (15)
	Left lower lobectomy			14 (19)
	Bilobectomy (right middle & lower)			3 (4)
	Pneumonectomy (left)			1 (3)

Lobectomy was performed in most patients (n = 67), and bilobectomy was performed in 3 patients, pneumonectomy in 1, and segmentectomy in 2. One patient underwent right middle lobectomy through same incision, 2 days after the right upper lobectomy, due to right middle lobe torsion.

Two patients (3%) needed the third port during the surgery because of severe pleural adhesion (Table 2), and suffered from prolonged air leakage after the surgery (Table 3). Conversion to thoracotomy was performed in 5 patients (7%) due to bleeding at pulmonary arterial branch (n = 3), anthracofibrotic lymph nodes around pulmonary artery (n = 1), and severe pleural adhesion (n = 1). Two of these 3 patients with bleeding at the pulmonary artery, had undergone neoadjuvant chemotherapy. Two of these 5 patients suffered from atelectasis or prolonged air leakage after the surgery.

**Table 2:** Characteristics of patients with conversion to 3 ports or thoracotomy during two ports VATS lobectomy

**Sex**	**Age**	**Diagnosis**	**Neoadjuvant chemotherapy**	**Cause of conversion**	**Type of surgery**	**Complications**
Conversion to three ports (n=2, 3.1%)						
Male	69	Metastatic lung cancer	Yes	Severe pleural adhesion	Right lower lobectomy	Prolonged air leak
Female	60	Primary lung cancer	No	Severe pleural adhesion	Left upper lobectomy	Prolonged air leak
Conversion to thoracotomy (n=5, 6.8%)						
Male	71	Primary lung cancer	Yes	Bleeding at pulmonary arterial branch	Right lower lobectomy	No
Male	74	Primary lung cancer	No	Calcified lymph node around pulmonary arterial branch	Right lower lobectomy	No
Male	71	Primary lung cancer	Yes	Bleeding at pulmonary arterial branch	Right upper lobectomy	Atelectasis
Male	59	Primary lung cancer	Yes	Bleeding at pulmonary arterial branch	Left upper lobectomy	No
Male	71	Primary lung cancer	No	Severe pleural adhesion	Right lower lobectomy	Prolonged air leak

**Table 3:** Postoperative complications

	**No (%)**
Prolonged air leak (>5 days)	4 (5.5)
Atelectasis	2 (2.7)
Pneumonia	1 (1.4)
Reoperation	1 (1.4)
Atrial fibrillation	1 (1.4)

The mean time of the operation, in 66 patients who were completed by two-incision VATS lobectomy, was 163.4 ± 30.40 minutes (range, 85~277). According to the site of lobectomy, the operation time of the upper lobectomy was longer than that of the middle or lower lobectomy (173.6 ± 32.51 minutes vs 154.3 ± 25.56, p = 0.010). Further, according to the period, the operation time of the first 33 cases was longer than the last 33 cases (171.3 ± 532.25 minutes vs 152.5 ± 32.72, p = 0.037). In 56 cases, which were completed by two-port VATS lobectomy for primary lung cancer, the total number of dissected lymph nodes per patient was 20.2 ± 11.2 (range, 6~52). The chest tube was removed on postoperative day 5.4 ± 2.8 (range, 3~15), and there was no occurrence of major perioperative morbidity and mortality.

## Discussion

Although major pulmonary resection using VATS has gained popularity, due to its potential benefits, which includes faster patient recovery, fewer complications, and shorter hospital stay without compromising the oncologic aspects of the operation, the definition of VATS lobectomy has not been established until now [5]. Various factors regulate the definition of VATS lobectomy, for example: use of rib spreading, variety of the devices, length and location of the utility incision, number of thoracotomy ports, scope of the light source or monitoring, how to treat vessels, how to dissect lymph node, and so on [16,17]. At the present time, most thoracic surgeons agree to define VATS lobectomy as a procedure with one 4 to 8 cm sized utility port incision and one to three 0.5 to 1 cm sized port incisions, while avoiding rib spreading, and with the use of a camera for viewing purposes [5,10,18].

In our hospital, since VATS lobectomy launched in March 2006, two ports and a utility incision had been used without rib spreading. As mentioned before, our method for port placement enabled the operator to only use the utility incision, and the assistant to only use the 5 mm port, most of the time during the operation.

While we had performed more than 120 VATS lobectomies, the need for the third port gradually has begun to decrease, with the build-up of the surgeon’s experience. In addition, around the same time, Borro and colleagues [15] published a paper on two-incision approach for VATS lobectomy. Therefore, we considered the two-incision VATS lobectomy feasible, and started to perform this procedure without the third port. To make the anatomical resection through only two incisions, it is essential to learn the technical tips. Firstly, the thoracoscope and the endostaplers are exchanged from one incision to the other for the resection of hilar structure, according to the lobectomy site [15]. Secondly, in case of the need for retraction of some structures, such as lung, bronchus, or vessel, during hilar dissection, and natural gravity could be used by rotating the operating table, or 5 mm sized blunt tip dissector (Ethicon Endo Surgery, LLC, 475 Calle C Guyando, PR 00969, USA) taken by the assistant, which could be introduced through the utility port.

When the 3rd port or conversion to thoracotomy was needed, we did it without hesitation during the surgery. Nevertheless, only in 2 patients, the 3rd port needed due to severe pleural adhesion, and in 5 patients (6.8%), conversion to thoracotomy was needed, mostly due to major vessel bleeding. The rate of conversion to thoracotomy in this study is comparable with the results of the Borro’s report [15] of two-incision VATS lobectomy (10%) and the standard VATS lobectomy (1.6%~11.8%) [17].

While controversy still exists concerning the oncologic effectiveness of VATS lobectomy, especially its efficacy in the mediastinal lymph node dissection (MLND), research to date has confirmed its feasibility and safety, as well as equivalent outcomes as compared to conventional open thoracotomy [19] Complete MLND has been the principle of our hospital, during VATS lobectomy in patients with primary lung cancer. When we began to perform the two-incision VATS lobectomy, we focused to keep this principle thoroughly. Therefore, since the first case of this procedure, the number of dissected lymph node has not been different from the data of our previous three-port VATS lobectomy or lobectomy through standard thoracotomy (20.2 ± 11.2 in this study, 22.1 ± 11.6 in our previous data including three-port VATS lobectomy or lobectomy through standard thoracotomy [20]). Our number of dissected lymph node is much higher than that of the Borro’s report (9.2 ± 5.4) [15] and it is comparable with the other group’s data of standard VATS lobectomy (10~23) [5,21-23].

The authors performed a two-incision VATS segmentectomy in 2 patients. We have considered patients with a tumor smaller than 2cm in diameter diagnosed or suspected as a clinical T1N0M0 carcinoma in the lung periphery based on a CT scan as candidates for the segmentectomy as an alternative anatomical resection [24,25]. They had no perioperative and postoperative problems, but we should have enough of these cases in order to compare with the conventional standard approach.

In this study, 2 patients with lung cancer who underwent neoadjuvant chemotherapy were included, although they are currently considered as contraindication for VATS lobectomy. Recently, as VATS lobectomy techniques continue to improve, expanding the indications for VATS, as well as for complex procedures, such as segmentectomy or sleeve resection, is currently a more pressing issue for treating advanced lung cancer [26]. Therefore, in our hospital, when it has seemed to be feasible to perform a two incision VATS lobectomy after careful thoracoscopic examination even in lung cancer patients with neoadjuvant chemotherapy, we have tried to perform this procedure.

The operation time in this study is similar to our previous data of standard VATS lobectomy (163.4 ± 30.40 vs. 146.8 ± 26.30 minutes, *p* = 0.238), and it is not different with Borro’ result (168 minutes) [15]. However, these results appeared to be longer than that of the standard VATS lobectomy in other series (118~130 minutes) [16,17]. When we analyzed this, according to the site of lobectomy, the operation time of upper lobectomy was longer than that of middle or lower lobectomy. Further, according to the period, the operation time of the first 33 cases was longer than the last 33 cases. Moreover, the operation time of the last 33 cases is not different from our previous data of standard VATS lobectomy (*p* = 0.526).

Therefore, we thought that as more procedures are performed and the learning curve of the surgeon improves with this two ports procedure, the operation time will become shortened and will therefore, have similar results as those recorded for the three ports approach. Two ports VATS lobectomy is not a new technique. It has been known that few surgeons have already carried out this procedure [15,27-29]. D’Amico’s group reported that two incisions were used in the majority of patients, among the 500 cases [27], and Borro and colleagures firstly used the terminology of two-incision VATS lobectomy [15]. The performance of two, three or four incisions in VATS lobectomy seems to have no influence on the short-term postoperative outcomes. Two ports VATS lobectomy is a consequence of greater skills acquired with experience [15], and could be a process of less minimally invasive procedure to a single port or natural orifice transluminal endoscopic surgery in the thoracic surgical field of the future. Therefore, as the accumulation of surgical skills and experience, we don’t need to insist on using the 3rd port in VATS lobectomy, although the elimination of the third port is not helpful to the patient in the operative outcomes.

## Conclusions

Two-incision VATS lobectomy is applicable in selected cases, and may obtain similar results with the conventional VATS lobectomy, given a certain period of learning curve.

## Abbreviations

CT: Computed tomography; NSCLC: Non-small cell lung cancer; VATS: Video-assisted thoracoscopic surgery.

## Competing interests

The authors declare that they have no competing interests.

## Authors’ contributions

HKK contributed to surgical procedure, reviewing data and drafting the article. HKS contributed to surgical procedure and analyzing data. HJL contributed to surgical procedure and collecting data. YHC contributed to reviewing data. All authors read and approved the final manuscript.
